# Veterinarians’ role in clients’ decision-making regarding seriously ill companion animal patients

**DOI:** 10.1186/s13028-016-0211-x

**Published:** 2016-05-25

**Authors:** Stine Billeschou Christiansen, Annemarie Thuri Kristensen, Jesper Lassen, Peter Sandøe

**Affiliations:** 1 Department of Large Animal Sciences, Faculty of Health and Medical Sciences, University of Copenhagen, Grønnegårdsvej 8, 1870 Frederiksberg C, Denmark; 2 Department of Veterinary Clinical and Animal Sciences, Faculty of Health and Medical Sciences, University of Copenhagen, Dyrlægevej 16, 1870 Frederiksberg C, Denmark; 3 Department of Food and Resource Economics, Faculty of Science, University of Copenhagen, Rolighedsvej 25, 1958 Frederiksberg C, Denmark

**Keywords:** Veterinary ethics, Paternalism, Autonomy, Informed consent, Shared decision-making, Euthanasia, Companion animals, Dogs, Animal welfare, Human-animal bond

## Abstract

**Background:**

When companion animals become seriously ill clients may have doubts about treatment choices, if any, and turn to veterinarians for help. But how should veterinarians reply? Influence on clients’ decision-making may or may not be acceptable—depending on one’s attitude to principles such as ‘paternalism’, ‘respect for autonomy’ and ‘shared decision-making’. This study takes as a starting point a situation where the animal is chronically ill, or aged, with potentially reduced animal welfare and client quality of life, and thus where clients need to consider treatment options or euthanasia. It is assumed throughout that both veterinarians and clients have the animals’ best interest at heart. The purpose of the study was to explore the challenges these situations hold and to investigate how clients experience veterinary influence. A second aim was to reflect on the ethical implications of the role of veterinarians in these situations. Qualitative interviews were conducted with 12 dog owners considering treatment or euthanasia of their chronically ill or aged dogs.

**Results:**

Challenges relating to the dog and to the client were identified. Some situations left the interviewees hesitant, e.g. if lacking a clear cut-off point, the dog appeared normal, the interviewee felt uncertain about treatments or animal welfare, or experienced conflicting concerns. Some interviewees found that veterinarians could influence their decisions. Such influence was received in different ways by the interviewees. Some interviewees wanted active involvement of the veterinarian in the decision-making process, and this may challenge a veterinarian’s wish to respect client autonomy.

**Conclusions:**

Different preferences are likely to exist amongst both veterinarians and clients about veterinary involvement in clients’ decision-making, and such preferences may vary according to the situation. It is suggested, that one way to handle this challenge is to include respect for client preference on veterinary involvement under a wider understanding of respect for autonomy, and to apply models of shared decision-making to veterinary practice. In any case there is a need to further explore the challenges these situations raise, and for the veterinary profession to engage in more formal and structured deliberation over the role of veterinarians in relation to clients’ decision-making.

**Electronic supplementary material:**

The online version of this article (doi:10.1186/s13028-016-0211-x) contains supplementary material, which is available to authorized users.

## Background

When a companion animal becomes seriously ill the client may be faced with difficult decisions. In some cases the answer is obvious. In others, however, the client may have doubts about which treatment to choose, whether to initiate treatment at all, or whether perhaps euthanasia is the better option. In such situations clients may turn to the veterinarian for help, asking ‘What would you do if it was your animal?’ Assuming that the situation leaves room for different lines of action, which are all in compliance with the law and can all be defended from a plausible ethical point of view, how should the veterinarian reply? Share his or her preferred option in similar situations? Offer an opinion on the best decision for the animal and the client? Inform the client of the options but refuse to offer any guidance on decision-making? Or initiate a dialogue with the client about what to do?

At a first glance ‘What would you do?’ is a straightforward question. Providing an answer backed by the relevant medical information may seem a relatively easy task. In reality, however, the situation often presents more complex issues. First of all, the circumstances of the veterinarian and client may differ, so the veterinarian’s preferred option may not be relevant to the client. Secondly, the client and veterinarian may have different ethical values, so what seems the right choice to the veterinarian may not be ethically acceptable to the client. It is, however, worth noting that the question may not really be about what the veterinarian would do, but rather be an appeal to the veterinarian to relieve the client of responsibility for the decision. But if the veterinarian feels that the decision needs to be made by the client alone, all he or she can and should do is to provide the necessary factual information which can serve as part of the basis for the client’s decision. Providing this information does, however, involve both a selection process (which information is relevant?) and communication with the client which may (unintentionally and non-verbally) convey the veterinarian’s personal views [1]. On the other hand, if the veterinarian engages in the decision-making, he or she may influence the outcome in a very direct way [2–6].

Veterinarians may have different ideas about how they should respond, which again may reflect different ethical points of view. For example, influence on a client’s decision-making may or may not be acceptable—depending on one’s attitude to ethical issues and principles such as ‘paternalism’, ‘respect for autonomy’, ‘informed consent’ and ‘shared decision-making’. In human medical ethics these topics have been discussed for a number of years. Paternalism may be understood as the view that it is acceptable to make decisions on behalf of patients with their best interests in mind. Respect for autonomy, by contrast, emphasizes that patients have the right to make their own choices. Truly autonomous decisions require the person to understand what the situation involves in order not to be manipulated. Respect for autonomy is therefore closely linked to the idea of informed consent. Informed consent, in turn, may be understood as given only if a person is competent to act, receives and comprehends full information about the options available, and voluntarily chooses one of those options. Together, respecting patient autonomy and obtaining informed consent thus aims at reducing the risk of undue influence on the patient’s decision-making. These principles have been widely debated within human medicine when it comes to guidance in doctor-patient relationships and decision-making. In recent years the paternalistic approach has been downplayed and instead patient autonomy and procedures for obtaining informed consent have been encouraged. Shifting the approach in this way, it is hoped, will better protect the patient’s right to decide on the care of his or her body and reduce the doctor’s risk of being blamed for carrying out procedures against a patient’s wish or interests (see [7–9] for thorough discussion of these principles). However, it has proved difficult to reach a consensus on the definitions of these key concepts and their applications, and the concepts have been challenged and have evolved over time.

Paternalism, where all decision-making is left to the doctor, and patient autonomy, where it is all left to the patient, may be seen as two extremes along a continuum of possibilities in the decision-making process. Intermediate alternatives, referred to as shared decision-making, may as a matter of fact reflect more accurately the way decisions are often made in practice. Shared decision-making is also discussed as an ideal—i.e. normatively, as the best way of handling the decision process in some cases [10]. Shared decision-making is described as a situation involving at least two participants, typically doctor and patient, who exchange information and preferences and reach an agreement on how to proceed. Agreement is understood as a readiness to proceed with a certain decision, not necessarily as agreement that this decision was the best option [11, 12]. Shared decision-making may be considered appropriate in cases when there are at least two reasonable medical options, and the choice between them is based on patient values and life circumstances [13].

The concepts of autonomy and informed consent can also provide guidance in veterinarian-client relationships. Inspired by human medical ethics, a similar development has taken place in veterinary medical ethics, where the ethical and communicative challenges, as well as the obligations veterinarians have when dealing with animals and clients, have been discussed. Issues include both how to respect client autonomy and how to handle conflicts when such autonomy poses risks to animal welfare. In a veterinary context, the situation differs from that of human medicine in several ways. Instead of normally just two parties to consider in the decision-making (doctor and patient) there are here three: the veterinarian, the animal and the client. The animal patient is the client’s property and is generally considered unable to participate in the decision-making. As a consequence legal issues have been raised, as well as concerns about decision-making on behalf of others, like those prompted by proxy decision-making on behalf of children and the mentally impaired [14–18]. Also, the perception of animals as unable to participate in decision-making has been challenged as animals can express preferences, and it has been argued that these preferences should be taken into account [17]. Concerns about autonomy and informed consent include considerations about the veterinarian’s obligations to the animal versus the client and issues arising from veterinary and client differences in assessing animal welfare [1–5, 17–33].

It has been argued, that veterinarians should promote client autonomy by providing information on the options and leaving clients to reach decisions even if the veterinarian finds the decision to be morally problematic [2]. One reason for this is for the veterinarian to avoid blame for the decisions made [2, 10, 26, 27]. Another reason is that the decision-making may give the client an opportunity for personal growth [34]. However, reasons have also been given in favour of a more active role. One relates to the protection of an animal’s welfare when the client is hesitating to make a decision that would end the animal’s suffering [1, 4, 29]. Another relates to respect for client preferences about the role of the veterinarian. Here, a client in doubt or emotionally overwhelmed may want the veterinarian to take an active role [1]. It has thus been argued, that the veterinarian-client relationship should be more of a two-way process [23].

Returning to the situation described in the beginning of this paper and the ‘What would you do?’ question, it ought to be obvious already that although this is well known amongst veterinarians in companion animal practice, there is no simple right way to respond, but rather conflicting principles underpinned by different reasons. Although a better understanding of the challenges such situations present would be helpful when supporting clients in decision-making, this scenario has received relatively little scholarly attention. Thus, one way forward is to take a closer look at situations where decision-making becomes challenging, and at the questions to which these situations give rise.

This study takes as a starting point a situation where the animal is chronically ill, or aged, with potentially reduced animal welfare and client quality of life. The client thus needs to consider treatment options or euthanasia, and it is supposed that both the veterinarian and client have the animal’s best interests at heart. The purpose of the study was first to explore the challenges these situations hold, and the way clients experience veterinary influence. A second aim was to reflect on the ethical implications of the role of the veterinarian in these situations.

This paper thus addresses three questions relating to situations when decision-making becomes difficult. First of all, in what kind of situations do clients find it difficult to make a decision on their own? Here the qualitative study identifies challenges for clients considering treatment or euthanasia of their chronically ill or aged dog. Next, in cases where the animal is neither suffering nor curable, how do veterinarians respond to clients’ hesitation to decide, and requests for advice? The qualitative study here analyses the way in which clients find that veterinarians have facilitated or influenced their decision-making. Finally, if a veterinarian takes a more active role, how should he or she engage in decisions about the initiation or continuation of treatments, or about euthanasia? Drawing on the results from the qualitative study, the role of veterinarians and the ethical implications when clients seek advice on treatment or euthanasia is addressed.

## Methods

In order to obtain detailed descriptions of a variety of client-experiences, individual in-depth qualitative interviews with dog owners were carried out. The dog owners were recruited among the clients of the University Hospital for Companion Animals at the University of Copenhagen, and a privately owned companion animal practice in Copenhagen.

Initially, searches were run in electronic patient records systems for diagnoses and choices of treatment that allowed potential interviewees to be identified. The results reported in this paper are part of a series of interviews which also concerned client quality of life. Both aspects relating to client quality of life and aspects relating to the sharing of concerns with the veterinarian therefore framed the selection of interviewees. Two initial recruitment criteria for the selection of dog owners for further screening were applied: (1) that the owner had engaged with treatments and care that could be expected to affect his or her own life; and (2) that where the dog had died, this was no longer than 8 months ago. Furthermore, the selection process focused on cases where notes were made in the patient records of clients expressing worries about animal welfare, treatment choices, euthanasia and/or their own quality of life, as these clients had obviously shared these concerns with the veterinarians.

The dog owners identified were then contacted and asked about their interest in, and consent to, participation and further screening. Given that this was a qualitative study exploring the different kinds of situation and challenge clients face when they need to make decisions about treatment and euthanasia, it was important to recruit a diverse group of interviewees. The purpose of the screening was therefore to ensure that interviewees were selected to cover, between them, a variety of factors that could reasonably be expected to influence the clients’ situation in various ways. Factors directly connected with the animal patient included diagnoses, medication needs and other forms of patient care, and the age of the dog and whether it was still alive. Client-related factors revolved primarily around the client’s circumstances and included composition of the household and the perceived economic burden of the veterinary treatment. The interviewees were thus selected with the goal of covering the diversity of the questions to be explored—not as a representative group reflecting the frequency, or distribution, of the relevant phenomena in clients in general [35]. Ten interviews had been conducted when only minor new nuances emerged. Two more interviews were then conducted to ensure that the point of saturation regarding the issues to be explored had been reached [36], i.e. twelve interviews were conducted in total. Results of the interviews regarding client quality of life and a table presenting the screening details and the diversity of the interviewees’ screening profiles have been published previously [37].

All interviews took place at the clients’ homes in 2008. In some households the legal owner of the dog was not its primary caregiver. In these cases, the primary caregiver was chosen for interview. The interviews were semi-structured and followed an interview guide. The interview guide ensured that all interviews would cover the same themes (although not necessarily in the same order), and also allowed interviews to follow issues that had emerged as important. The themes of the interview guide that were relevant to this paper were: client concerns about animal welfare and euthanasia (with the aim of uncovering the kind of concerns experienced and challenges faced in client decision-making); and client experiences of veterinary visits (with the aim of uncovering how clients found that veterinarians had or could have helped them make decisions). The questions in the interview guide were open so as to stimulate fuller replies, which were explored further: for example, ‘Did you worry about anything?’ and ‘Do you think the vet did something to help you through this?’ (The interview guide is available as Additional file 1). Beyond what was requested by the interviewer, interviewees were encouraged to add information they considered relevant. The interviews lasted 2 h on average. They were recorded and transcribed verbatim.

The transcribed interviews were analysed qualitatively by the first author using the software ATLAS.ti 5.0 to code and retrieve relevant text. The construction of codes and initial data analysis followed the template organizing style [38], with codes based on key points from the interview guide as well as additional aspects raised in the interviews. The text relating to each code was then analysed for features illuminating that aspect: for example, for the code ‘euthanasia decision’ issues relating to both challenges of the situation and to decision-making then emerged, which in turn triggered a closer examination of the aspects that made it harder or easier to make decisions about euthanasia. Abductive reasoning, allowing dynamic interaction between data and the generation of ideas [39], thus inspired further analysis and interpretation.

The study was approved by the Ethical and Administrative Committee of the Department of Veterinary Clinical and Animal Sciences, University of Copenhagen, as well as by the Danish Data Protection Agency. Interview excerpts appearing in this paper were translated from Danish by the first author. In some cases text has been omitted, which is indicated by “…(…)…”, and text in […] has sometimes been added for clarity.

When reporting the results and considering the ethical implications the term “interviewee” is used when reference is made to the interviews in this study, and the term “client” is used for general discussion. The term “owner” is used in the short dog-owner description placed at the end of each interview quote.

## Results

The interviews generated data informing the two questions aimed at the study’s first purpose: exploring the situation of the client when he or she is considering further treatment or euthanasia of a chronically ill or aged dog. The first question explored the challenges these situations hold, i.e. what features of the situation ease or complicate the client’s decision-making? The second question explored the clients’ experiences of veterinary influence, i.e. how do clients find that veterinarians have, or could have, facilitated their decision-making? It should be noted, that in some cases the experiences shared by the interviewees related to veterinary practices other than those from which they were recruited. The second purpose—ethical reflection on the role of the veterinarian in these situations—is dealt with in the discussion.

### Features easing or complicating the client’s decision-making

For some interviewees the decision to euthanize the dog was easy, relatively speaking. For others, it was more difficult. Here the challenges related either mainly to the dog or mainly to the client.

### Situations leaving interviewees capable and at peace with their decisions

Some conditions made the decision to euthanize the dog easier. Several related to poor welfare of the dog: for example, the dog was suffering, no longer happy, unable to function, or otherwise clearly not doing well; or episodes of illness were occurring too often. Other conditions related to the exhaustion of other options, i.e. when everything had been tried out and it was futile to pursue treatment. Clear signs of health deterioration or client fatigue tended to favour the decision to euthanize. More than one of these conditions could be present at the same time. Thus:

“It was the last week or so that it started going downhill, and I’d already asked if there was anything else we could do…(…)…And when there wasn’t anything left to do and we could just anticipate her getting worse and worse, I felt that then it wasn’t as hard to make that decision. Because it didn’t work… *[It worked*] neither for the dog nor for us” *(owner of euthanized dog with cancer).*

### Situations where interviewees found decision-making more difficult

Other situations created more of a grey area and interviewee hesitation. Here dog-related challenges included the lack of a clear cut-off point in cases with slow deterioration or fluctuating levels of health. Client-related challenges included lack of knowledge, conflicts of concerns and the taking of responsibility.

#### Dog-related challenges

##### Slow deterioration

A clear cut-off point was hard to identify when the dog’s condition was slowly deteriorating without any major turn of events. It could be difficult to decide exactly when to stop, as the lack of a clear sign, well-defined criteria or an obvious cut-off point, could make it hard to comfortably draw a line and opt for euthanasia. For example:

“I think it is difficult to know when to make that decision, because, for about one and a half years she has been old and you’ve been saying now is the time you should consider getting it done. But I have to admit that I also think that if you are not in pain, if you can move around in the garden more or less without being anxious or afraid or such like, when you can find your water and food and you can eat and are not incontinent, and can do your business and accept patting …then I don’t have to make the decision. But exactly when you should do it, that is a bit difficult, I think” (*owner of euthanized old dog with dementia).*

##### Fluctuating levels of health

When good health alternated with bad, a clear cut-off point was also lacking, but another challenge was added when it came to deciding that enough is enough. During the bad episodes considerations in favour of ceasing treatment might emerge, but then questions might also arise why euthanasia was not chosen last time and why the dog should not be given yet another chance. Some interviewees felt torn between blaming themselves for not having euthanized the dog earlier to avoid this phase, on the one hand, and hoping that this episode would be the last and that the next course of treatment would make things better, on the other. As some conditions allow repeated treatments and perhaps also hold a seemingly endless list of additional treatment options, interviewees could find themselves caught in a pattern where they had very strong hopes that things would get better, and thus kept seeking treatments, optimistically believing that the next one would be the one that would get the condition under control. For example:

“It is just really hard to say, ‘Okay, now it is over’, because….I mean, it’s just, why now? I could allow for one more episode or I could have stopped a long time ago, but when you have gone down the road of wanting to try everything for him, it is just really difficult suddenly to put your foot down and say this is it” *(owner of euthanized dog with epilepsy).*

In addition, if the dog appeared normal, and was, for instance, playing happily in the garden when the euthanasia decision was close, it could be hard to bring the dog to the veterinarian for the final visit, even if the interviewee knew that the dog was ill and had witnessed episodes when the dog was obviously not well:

“You feel like you walk in with a dog that you can’t see or sense anything wrong with, so it makes it a bit hard to euthanize it, when you can’t really see that it is ill. But I knew that he was….” *(owner of euthanized dog with epilepsy).*

#### Client-related challenges

##### Lack of knowledge

Knowledge was a significant factor in the decision-making process. For example:

“If we had known even more about it, it would probably have been easier to make the decision than it was” *(owner of living dog with cancer).*

But, although finding that veterinarians took time to provide information, the interviewees could feel unable to absorb or understand all the information they had been given about the dog’s medical condition, and thus doubt their own judgements about the best way forward. For example:

“I think they were really good at taking time to explain if you had any questions, and they also told us a lot about the disease, but to remember exactly what happens, it was…(…)…I don’t know if I actually got any wiser about what goes on during such a treatment…(…)…I had to trust that it was good, what they told me to do, and that was what I had decided to do, I think” *(owner of euthanized dog with cancer).*

Strong concerns about animal welfare were expressed along with the need to ensure that the dog was not suffering. But unless their dogs showed clear signs of being well or unwell, interviewees sometimes felt uncertain about their dogs’ welfare. For example:

“If only you knew, he is old and he is tired, but he must not feel bad, and I just can’t tell, and maybe it’s because I close my eyes, because maybe there is small chance, right?” *(owner of living old dog with diabetes)*.

Uncertainty about the animal patient’s welfare raised concerns about when it was time to consider euthanasia:

“I don’t know if you can measure whether a dog is in pain or not…(…)…but it would be nice if you could be more certain that now it is time to do it, because now it is better for the dog” *(owner of euthanized old dog with dementia).*

##### Conflicting concerns

In the process of decision-making, weighing up conflicting concerns about the dog and the interviewee could be difficult. Several kinds of conflict arose when treatments and euthanasia were being considered. One set of conflicts related to the treatment of the dog only. Here, the wish to give the dog a chance by initiating or continuing treatment was in conflict with the wish to protect the dog from suffering and thus choose euthanasia. When a treatment was chosen, conflicts could arise because the desired effects of the treatment had to be weighed against unwanted potential side-effects. For example:

“I also considered an operation to insert a tube in her throat so she could breathe. Because that was the only operation they could offer, and considering that there was a 50 % chance, I wanted to give her the 50 %, but I really wouldn’t put her through all that. The little dog had been through enough. So it wasn’t worth it” *(owner of euthanized dog with asthma)*.

Another set of conflicts put dog interests against human interests. The risk of postponing euthanasia for too long for the interviewee’s own sake (e.g. because it was hard for the interviewee to part with the dog) was one issue of this kind. Others focused on finding the right balance of sacrifice made by the interviewee when caring for the dog, and on the concern that euthanasia should not simply be an easy way out of commitments to the dog:

“It shouldn’t be euthanasia just because it is hard for me. That’s how I feel. I don’t want to euthanize it just because it is hard for me. And … I think it sounds bad, too, to euthanize an animal just because it does not fit in. I could never do that” *(owner of living dog with epilepsy).*

Other conflicts arose when humans identified with the dog’s condition. Here euthanasia could be postponed as it raised complex questions, too tough to deal with, about human patients in similar situations. An example:

“My husband was ill and he found it very difficult that we should part with the dog, because he thought, well, now she is ill and old and then you just get rid of her, and maybe he felt it was a bit like that with him too, you know” *(owner of euthanized old dog with dementia).*

Finally, conflicts sometimes arose when family members disagreed over the choice of treatment or about the timing of euthanasia.

##### Taking responsibility

Taking responsibility for ending the dog’s life and the feeling of being an executioner were hard for some interviewees to bear. It could be seen as preferable for the dog to die peacefully in its sleep, or even as a result of an accident, as this would at least relieve the interviewee of responsibility for the death. Although acknowledging that decisions about euthanasia were their responsibility, making this decision sometimes felt wrong and made the interviewee feel like a bad person for taking a life:

“It would be easier if they were hit by a bus or something, I think. Because then it’s over, or if you just woke up one day and it was lying there, dead. But that you have to make that decision yourself. I think that’s horrible” *(owner of living dog with allergy)*.

### How clients find that veterinarians had or could have facilitated their decision-making

The interviewees generally expressed a lot of concern about their dogs’ welfare, and they were aware that in some cases inaction could potentially lead to the one situation they probably feared the most: one in which they had let their dogs suffer. Where there was doubt about whether to euthanize the dog, two different strategies were reported. One was: better to be safe than sorry. Here it is acknowledged that the only way to ensure that the dog does not suffer is euthanasia at a relatively early stage. But at the same time it might seem pointless to euthanize the dog while it is still well simply to avoid the risk of suffering:

“That would not make sense. Well, you better get it over with right away, it will be hell over the next 8 months – don’t go there! Then you would be in a situation where you put down an animal that was doing fine and all. And I don’t know, I mean, I don’t think that is okay either” *(owner of living old dog with dementia)*.

The other strategy was to ask friends, family or veterinarians for opinions and help with the decision to be made. Such input, particularly from the veterinarian, could be instrumental in moving the decision-making process forward.

Interviewees gave two main reasons for wanting more involvement from veterinarians in decisions about treatment and euthanasia. The first was the feeling that they could not assess the dog’s condition, or the implications of the treatment, properly; they felt they needed the veterinarian’s expert opinion before making a decision. The second was that, particularly where euthanasia was concerned, they felt that the responsibility of this decision was too great for them to take on alone. The opportunity to share the decision-making, and thus the responsibility, with the veterinarian brought relief here.

#### How veterinarians influence decisions

Interviewees reported several ways in which veterinarians had contributed to their decision-making: by providing information and professional assessments, by supporting impending decisions, by legitimising certain considerations, and by providing specific guidance.

##### The provision of information and professional assessments

When interviewees found that they did not have the required knowledge to assess their dog’s welfare they sometimes relied on the veterinarian’s evaluation. The interviewees explained how the veterinarian could provide them with the relevant information on the dog’s medical condition and the treatment options; and he or she could offer a professional assessment of the situation and the dog’s welfare:

“I spoke to the vet and the vet presented the arguments – this thing about [*the fact that*] it had not spread further yet, it was only in one lymph node, and we had come in at an early stage, and that he wasn’t older than he was, and that despite this being an aggressive form it would make good sense to start the treatment” *(owner of living dog with cancer).*

At the same time, interviewees could be aware that veterinary knowledge covered only the medical aspects of the illness and treatment, and physical aspects of dog welfare. In the assessment of the dog’s mental state and functioning in its everyday life, an interviewee might well feel more competent than the veterinarian. It was mentioned that the overall assessment of the dog’s welfare was best achieved through a combination of veterinary and client knowledge. For example:

“They know much more about the dog somehow, about the physical stuff at least, than I do, but I know more about the emotional issues than they do; they don’t know my dog like I do. I think it is important to mix both” *(owner of living dog with epilepsy).*

##### Support for impending decisions

Where an interviewee was leaning towards a particular decision, such as to euthanize the dog, confirmation from someone else that this was the right decision was sometimes sought in an effort to move the decision-making on. In this situation arguments in favour of continued treatment could be less welcome. For example:

“But I still needed confirmation that this [*euthanasia*] was right, and he [*the interviewee’s brother*] didn’t at the time, and therefore he shouldn’t talk about it then. But the veterinarian confirmed straight away that this was the right thing to do” *(owner of euthanized dog with asthma).*

Hope was also expressed that the veterinarian would not suggest further initiatives, but in cases where the veterinarian was reluctant to euthanize, an impending decision to do just that might be reconsidered:

“Clearly I need advice, but not if I take him to a vet who says I think we should give him some pills and wait and see for 10 days. Then I honestly don’t know what to say, because I don’t think I want that…(…)…But I know what the vet will say, that is, that the vet will support that my dog should be euthanized…(…)…If the vet surprises me and says, well, your dog should have some antibiotics, I think that, because it is this vet, I will say ‘OK, fine then, that’s what we’ll do’, because I trust this one” *(owner of living old dog with dementia).*

##### Veterinary legitimisation of certain considerations

Certain considerations could be difficult to address for or may perhaps be suppressed by the interviewee. Such considerations could, however, be decisive either in favour of, or against, a certain course of action. Some interviewees reported that the veterinarian’s sensitivity to such considerations could be important for their decision-making. For example, if the thought of euthanasia was associated with a feeling of guilt, it could be difficult to address this issue with the veterinarian, and a veterinarian engaged in a longer treatment process might on occasion seem less sensitive to a wish for dialogue about the euthanasia option. Thus, for example:

“I tried to bring up that, maybe, I was considering euthanasia, and it was really difficult for me, because I think it is wrong, I feel I’m a bad person because, well, my dog is my child, right … It was as if they didn’t really understand what I was trying to say” *(owner of living dog with epilepsy).*

Furthermore, interviewees might neglect the impact on their own lives resulting from their dog’s needs for special care and thus jeopardize their own quality of life. It was reported how a veterinarian could legitimise concerns for the interviewee’s own well-being by raising awareness of concerns and limits to further treatment other than those relating to the dog, e.g.:

“The vet reminded me that it was … well, that it was not a decision I should feel bad about if I decided to euthanize my dog if I felt he had become too much for me. And I think it was good to get that part in as well, because I felt like I’d totally forgotten about that, because you just take care of this animal like you’ve always done and all…” (*owner of living old dog with dementia).*

##### The provision of guidance

In some cases interviewees reported that the veterinarian had tried to bring the decision-making forward, e.g. by recommending euthanasia or giving the interviewee a deadline for final evaluation. These interviewees were unsure if they would themselves have made the decision at that particular time, but found the encouragement from the veterinarian helpful, as part of the responsibility was taken off their shoulders:

“They were the ones who said enough was enough. And then I thought that, yeah, it probably is. So I agreed that now was the time – I could see she got worse and worse the last week, you know. I mean, if she had been bad before, hopefully I would have made that decision before, because she shouldn’t be. So, I believe it was right, what they said, but I’m not quite sure if I myself would also have said it at that time too” *(owner of euthanized dog with cancer)*.

Similarly, a continued support for treatment by the veterinarian could be perceived as an indication that euthanasia, based on welfare concerns, was unnecessary:

“The vet wouldn’t just say we should go on and on if it wasn’t right for the dog” *(owner of living dog with cancer).*

#### Client perspectives on veterinary influence

In cases where interviewees had shared their doubts about treatment and euthanasia with the veterinarian three separate perspectives were found. One perspective was simple awareness that the veterinarian had opinions based on concerns other than just a clinical evaluation without this necessarily leading to a feeling of being influenced in the decision-making. For example:

“The vet has expressed an ethical opinion, which is fine with me. I mean, we don’t have to agree about how we see things, but I want to know what the vet’s opinion is. And then I can relate to everything else the vet says” *(owner of living old dog with dementia).*

Similarly there was awareness that the veterinarian might have his or her own interests or agenda:

“I also knew that maybe the vet was not completely unbiased in relation to a wish to get going and get some experiences. But what the vet did say was that it was a good decision, that we had chosen a different treatment, and the vet really supported it” *(owner of living dog with cancer).*

Another perspective involved the interviewee striving to be autonomous in the decision-making, yet acknowledging some veterinary influence on decisions made. When the interviewee felt unable to assess the dog’s condition or the implications of a treatment properly, a veterinarian’s opinion could be seen as necessary in order to decide, even though this asymmetry in knowledge could influence the interviewee in the decision-making process:

“I accepted the treatment anyway. I found I couldn’t do anything else – I don’t know these things, so I had to let them … well, the decisions were of course my own, but it is really difficult to decide, because you don’t have the knowledge needed. So, even though they were very keen about the decision being mine and tried to inform me, I think it is still difficult to work out. So, it is more like, we will do what you believe in, because I don’t really have the ability to decide one thing or the other – that’s the way it is” *(owner of euthanized dog with cancer).*

In the third perspective there was a desire for greater veterinary involvement, or simply for straightforward reliance on the veterinarian’s opinion, although these interviewees also accepted that decisions were ultimately theirs. These interviewees wanted clearer guidance in decision-making, in particular concerning euthanasia. One factor in this was difficulty assessing animal welfare. Thus:

“I’m not a vet. I have no tools to assess how he is. I can seek help and have someone look at him and tell me…(…)…because how can I assess if it’s okay anymore? Have we gone too far? That’s what I’m afraid of, and it would be easier for me if someone said now you need to make a decision” *(owner of living dog with allergy).*

Another factor was a feeling that the responsibility of the decision to euthanize was too big to bear alone, and thus the belief that the decision needed to be made together with the vet:

“The veterinarian has to tell me [*when euthanasia is advisable*], because I think it is a bit much for me to take on” *(owner of living old dog with dementia).*

This stronger kind of involvement by a veterinarian was sometimes seen in a positive light. Thus:

“It was quite a relief to leave the responsibility to the vet who said now you have a two-month deadline. And then I thought, ‘Oh, that’s nice, now I don’t have to worry about that anymore’ – I mean, when to euthanize him. Because I knew he had to be euthanized at some point. I just didn’t know how far to go…(…)…so it really was a relief when the vet said that. Then it was like the vet’s decision or responsibility, even though in the end the decision was of course mine” *(owner of euthanized dog with epilepsy).*

Thus, where interviewees felt uncomfortable about making the right clinical or ethical judgement, or unable to do so, the influence of the veterinarian could be significant – both in terms of what decision was made and when. Moreover, the veterinary influence sometimes provided emotional support to interviewees in handling the challenges they faced.

## Discussion

There are few actual studies on the veterinarian-client relationship, although much literature mentions the importance of this topic (see [40] for a review). This study is (to the authors’ knowledge) one of few to address the challenges in decision-making. Sanders [41, 42], Pilgram [40] and Morris [32] primarily explored the veterinarian’s perspective on veterinary-client relations, and they covered several issues of relevance to the decision-making process. This study reports the challenges as experienced from a client perspective, and more specifically, what clients encounter when they are faced with treatment choices and the possible euthanasia of their dog. This study shows that some situations left interviewees feeling hesitant in their decision-making. While some challenges mainly related to the dog’s medical condition, others related to the interviewee. Veterinarians could facilitate and in some cases influence the decision-making in several ways. The interviewees expressed different perspectives on this—one being a wish for the veterinarian to be (more) involved in the decision-making. These results are taken as a starting point for further discussion on the ethical implications for the role of veterinarians, when clients are faced with difficult decisions.

The interviewees were selected for this qualitative study, and the results therefore allow conclusions only about clients with dogs and with profiles matching the criteria of selection. Details of these clients’ strong sense of obligation and commitment to care for their dogs have been described elsewhere [37]. Clearly, the general population of clients is much more diverse. Furthermore, not all findings apply to all interviewees. The circumstances and the interviewees’ experiences differ, or the interviewees may respond differently to the challenges they encounter. However, the results can be seen to demonstrate that decisions about treatment choices and euthanasia are more difficult in some situations than others, and then to provide an overview of the nature of such situations and the various ways in which interviewees experienced or wanted veterinary influence on their decision-making.

The interviews were conducted in Denmark, and it may be worth noting two points in relation to the legal framework in this country. Firstly, according to the Danish Animal Welfare Act veterinarians have a legal obligation to intervene if they are aware of an incurably ill animal that will experience unnecessary suffering if it is allowed to go on living, e.g. to ensure that such an animal is euthanized, even if this goes against the wish of the client [43]. Secondly, there is no legal structure to guide or secure how to get informed consent from clients. None of the interviewees reported any conflicts in relation to these legal issues, though. Cultural differences, if applying these results beyond Denmark, could be worth to consider as human-animal relations may be sensitive to cultural context [44]. Also, experiences may be different for clients with other companion animals than dogs. The experiences shared in this study are, however, assumed to be similar to experiences of clients with dogs in other Western countries, who have the animal’s best interest at heart and are facing difficult decisions about treatments and euthanasia. It should be noted, though, that only the interviewees’ perspectives on this matter was included, and understandings of the concept of best interest were not explored.

It should be noted as well that the interviewees were in rather different phases in terms of time since their dog’s diagnosis, whether their dog was still alive or had been euthanized, and—in cases in which it had been euthanized—time since the euthanasia was performed. Animals may adapt [45], and clients’ perceptions and priorities may change with time and circumstances [46, 47]. The interviews reflected only the interviewee’s situation at one specific point in time. Additional interviews with the same individuals at different stages in the process would have provided a fuller picture. The results thus do not provide in-depth information on the decision-making process itself.

The overall picture in this study of the difficulties and influences a client with a seriously ill or aged dog may experience is, however, still believed to be robust: that is, it sheds some light on this complex situation and can be used as a basis for further ethical deliberation.

### Ethical considerations

One of the key factors in decision-making identified in this study is knowledge—about the disease, the treatment options, and animal welfare. The study showed that veterinarians play an important role as providers of such knowledge. This raises questions about what knowledge is relevant if the client is to consent to a procedure on an informed basis, about who can provide what kind of knowledge, and about what level of detail is sufficient.

The experience of veterinary influence described in this study, in turn, raises two points. The first is that veterinarians may influence decisions even when they make an effort not to do so. The second is that clients may want veterinary influence on decision-making. These points call for a closer examination of the notion of (respect for) autonomous decision-making and how decisions can be shared in practice.

Informed consent, autonomy and shared decision-making will therefore be examined more closely in light of the study’s results, with a focus on the ethical issues concerning the relation between the veterinarian and the client. No attention will be paid to situations in which client autonomy can be disregarded because animal welfare is at risk. The making of decisions by proxy may, of course, give rise to a special obligation to make decisions believed to be in the interests of the animal patient in question. As no conflicts appeared, in this study, to jeopardize the interests of the animals in the decision-making, this issue will not be considered further. Nor will legal aspects be addressed.

### When is informed consent, or informed choice, sufficiently informed?

Although efforts are made to present relevant information at a level of detail suited to the client’s understanding, the provision of this information does not necessarily mean that the client fully comprehends the details of the treatment options being explained [25, 33, 48]. The use of consent forms in veterinary practice has been encouraged [3], but it has been pointed out that ensuring that the client is properly informed is a vital part of informed consent [27], and that a signed consent form is not necessarily evidence of informed consent [49]. In this study, although the interviewees had made their decisions themselves and had agreed to procedures, it did not automatically follow that they truly understood all of the implications at stake.

The issue of informed consent is complex, but the objective of informed consent is that clients are provided with adequate information so they can make the right decision for their animal and for themselves [25] (see [27] for a thorough discussion on informed consent). It has been argued that consent can never be fully informed, and that therefore what should be aimed for is relevantly informed consent [7]. But what is adequate informed consent [25] and what information is relevant? Clearly, veterinarians need to keep up with medical knowledge to provide the necessary information to clients [50]. But in some cases there may be limited relevant information about the medical condition and treatment which can make the disclosure task difficult for the veterinarian and require that the veterinarian also informs the client of uncertainties regarding the treatment [28]. Follow-up studies may assist veterinarians [51–53], but if he or she consults follow-up studies, the studies will typically cover clinical aspects such as treatment success and side effects, but not necessarily all relevant aspects of animal welfare (including such matters as the animal’s mental state and functioning in everyday life) [51, 53]. In addition, the veterinarian will not necessarily be fully aware of the potential impact, on clients’ lives, of caring for their ill animals [2, 37]. However, the veterinarian needs to address these issues too if the client is to be fully and relevantly informed about what a treatment option entails.

The information from clients complements the veterinarian’s assessment of animal welfare [17, 33, 54–59]. The present study indicates that clients may in some cases feel better prepared than their veterinarians to assess certain aspects of their animal’s welfare because they know their animals better and see how they are doing in everyday life. This has been argued by others too [1, 17], although it has also been pointed out that veterinarians may be better placed to assess animal welfare in other cases and are better informed about likely prognoses [17]. All of this suggests that an exchange of the veterinarian’s and the client’s knowledge could be the best way to move the decision-making forward.

Given that clients may not fully comprehend all of the clinical information and its implications for their animal and themselves, and given also that such information may not always be available, one could argue that the client’s consent or choice—given more options—is in practice probably rarely fully informed. Perhaps a less ambitious level of information in informed choice and consent is more realistic in an everyday scenario. It may not be practically feasible to present all treatment options to clients [28], and it has been suggested that veterinarians should only offer reasonable options [33]. Furthermore, it may be argued that veterinarians should consider how much clients want to know [3, 50], and that clients do not need to understand every nuance of a proposed treatment, but the information should be sufficient for the client to reasonably make informed decisions [27]. Thus, it may suffice that all available (and reasonable) options and known implications have been presented, and that the client feels sufficiently informed and agrees to the suggestions being made. It still needs to be determined, however, what is considered reasonable.

### When is an autonomous decision sufficiently autonomous?

Full autonomy in decision-making may be challenged first by the limitations of knowledge just mentioned; and second, because of the worries, doubts and sadness clients may be facing at the time of decision-making, leaving them vulnerable to influence from an authority such as the veterinarian. In human medicine it has been argued that the right to choose does not mean a duty to choose [8, 13], and in fact when they are ill it may be a relief to patients not to be making demanding autonomous decisions [7]. It appears that similar considerations, about the need to make decisions in a state of mental distress, may be relevant in veterinary medicine. This study showed that for some interviewees the influence of a veterinarian was instrumental in ending the dog’s life at a time which seemed suitable to the interviewee in terms of dog welfare and in giving the interviewee some peace of mind. In addition some interviewees had appreciated the way the veterinarian had helped them bring an end to a seemingly endless series of procedures that brought little if any improvement and ultimately only postponed the inevitable.

But to what extent is this compatible with the ideal of respect for client autonomy? It has been argued that human patients should be fully entitled, but not required, to take an active role in decision-making [60]. It has also been pointed out that there are two levels of respect for autonomy: one demands respect for the patient’s wish for the doctor to be involved in decision-making, the other requires respect for the patient’s wishes regarding treatment [61]. One could then argue, in the veterinary context, that client-autonomy is respected if and only if both levels are considered, i.e. when a client feels that the wish for veterinary involvement as well as the possibility to choose or reject a certain treatment or euthanasia is respected. This will allow the client to delegate some (or all) of the decision-making to the veterinarian, yet continues to give the client the option to veto a decision if he or she considers it unacceptable.

### Is shared decision-making the way forward?

The findings in this study support the understanding that both clients and veterinarians are sources of relevant information, and that some clients would like greater involvement in decision-making. This suggests that the concept of shared decision-making may be the way forward in some cases.

In human medicine, it has been shown that patients may have different preferences regarding the involvement of doctors in decision-making—that some want to make their own decisions, some want to share the decision-making, and some prefer to delegate responsibility to the doctor [11]. The patient’s personality may also be a factor to consider, and shared decision-making, therefore, is not the best solution in all encounters [62]. In fact, the whish for shared decision-making will depend on the context—e.g. on the disease and treatment options in question—and participation may be more desirable in situations when there is no clear best option [63]. Similar observations have been made in a veterinary context, where clients may have different preferences about the veterinarian’s role in decision-making [64], and different approaches may be appropriate depending on the situation [33]. These observations support the findings in this study, that in situations when there was no obviously best option, where interviewees felt hesitant about making decisions, veterinary involvement could well be both sought and appreciated by the interviewee.

Shared decision-making is not, however, a simple task in practice. Several issues have been identified in both human and veterinary medicine. Different roles have been identified: two such are the facilitative role, where the doctor/veterinarian helps the patient/client uncover what he or she considers the best option, and a more collaborative role, where the doctor’s/veterinarian’s own preferences are drawn into the decision-making process [64, 65]. Furthermore, the same doctor/veterinarian may assume different roles in different consultations and their roles may even change during one and the same consultation [12, 32, 65]. In addition, it has been pointed out that wanting to participate in decision-making may be different from wanting to make the final decision [66]. The risk of patients/clients being pulled away from their own preferred decisions under the influence of doctors/veterinarians has also been emphasized [27, 67, 68]. Patients/clients may in such cases find that the doctor/veterinarian has more information than they do, or they may feel insecure about taking on the responsibility of decision-making [20, 64, 69]. These concerns are very similar to those expressed by interviewees in this study.

Potential disagreements may also become an issue. The interests of the animal and the client are not always reconcilable [70], and the veterinarian may have his or her own interests [4]. Disagreements may arise over e.g. the welfare and value of an animal, responsibilities to animals, and what the role of the veterinarian should be [5, 32, 41]. Veterinarians may not be willing to leave the decision entirely with clients, as they want to protect the animals’ best interest (from their point of view), and if the client requests something that the veterinarian does not agree with, the veterinarian may try to negotiate a different course of action for his or her patient [32]. End-of-life care and euthanasia decision-making can be influenced by the ability of those involved to reach consensus [71], and veterinarians thus need to handle disagreements when engaging in shared decision-making [32]. It has been suggested that perhaps veterinary practice can learn from human medicine, e.g. regarding the development of decision-aids [33].

In human medicine patients often want to share decision-making with their doctor [72]. In the veterinary context getting information about the client’s perspective may promote shared decision-making [73], but some have warned against taking the principle of informed consent lightly just because clients ask for veterinary involvement [27]. Others suggest that veterinarians should inform clients of their personal view, and thus help clients even though they then risk influencing decisions made [31]. Some have argued that providing comfort for the human caregiver is a legitimate responsibility and goal in veterinary practice [74], and that it is acceptable to influence clients when they explicitly want to be influenced and animal welfare is respected [1]. Shared decision-making thus seems to offer valuable perspectives on how veterinarians can engage in decision-making, but several challenges still need to be addressed.

### So, what should you do?

In addition to being a medical expert, the veterinarian may act as a support person and facilitator in decision-making [20, 42]. Veterinarians may find that they need to offer emotional as well as informational and practical support [40], and clients may indeed expect this [75]. However, veterinarians may disagree with their clients about the best way forward [76], and professional codes of conduct may be insufficient to guide veterinarians on matters such as euthanasia and end-of-life issues [77]. There may thus be a need for more emphasis on skills in communicating with clients with terminally ill animals in the veterinary education [78], and the ethical issues addressed in this paper may assist development of useful frameworks.

In human medicine it has been argued that the focus of medical ethics should be on respect for the wishes of the autonomous person, rather than respect for the autonomous choices [9, 79], and it has been suggested to ask patients first who should be involved and how decisions should be made, before obtaining informed consent regarding what decision to make [72]. Similarly, when addressing treatment options requiring difficult decisions the veterinarian could explore what level and kind of involvement the client wants in the decision-making. Next, the role of the veterinarian could be to follow these wishes until a decision on treatment has been made, keeping in mind that the client’s attitude to veterinary involvement, or the client’s preferred treatment option, may change in the process.

Perhaps a specifically designed consent form should be created, to clearly acknowledge that such agreements have been made. This way the client could get the help in decision-making asked for, hopefully without the veterinarian risking the criticism that he or she has failed to respect the client’s own wishes in the decision-making.

The approach of shared decision-making does, however, raise additional issues. Veterinary influence on a client’s decision-making is sometimes unavoidable. How can such influence be made transparent? And how can the risk of the client being moved in a direction that goes against his or her own considered preferences be minimized? Finally, the idea of shared decision-making presupposes that both the client and the veterinarian agree to participate. Therefore it is not enough that the client wants the veterinarian to be involved in the decision-making; the veterinarian also has to be willing to take on this role in order to produce the best outcome. The notion of shared decision-making, including the associated ethical issues, and a general development of the ethical framework in veterinary decision-making clearly deserve further attention.

## Conclusions

This study showed that clients may encounter several challenges when they are faced with treatment choices and the possible euthanasia of their dog, and in some cases they want the veterinarian to be involved in the decision-making. The reflections presented on key ethical principles relating to clients’ decision-making, suggest that there is a need to further explore the challenges these situations raise, and to develop the ethical framework around the role of veterinarians in clients’ decision-making. The idea of shared decision-making deserves special consideration.

## Additional file

**Additional file 1.** Interview guide for interviews with dog owners caring for aged and ill animals.
