# Supplementary material for: Veterinarians’ role in clients’ decision-making regarding seriously ill companion animal patients
Source: Acta Vet Scand. 2016 May 25;58:30. doi: 10.1186/s13028-016-0211-x (PMC4879734; doi:10.1186/s13028-016-0211-x)
Supplement: Supplementary file 1 — Additional file 1. Interview guide for interviews with dog owners caring for aged and ill animals. [file 13028_2016_211_MOESM1_ESM.pdf]

Christiansen SB, Kristensen AT, Lassen J & Sandøe P. Veterinarians' role in clients' decision-making regarding seriously ill companion animal patients. Acta Veterinaria Scandinavica, 2016. doi:10.1186/s13028-016-0211-x

Additional file 1: Interview guide for interviews with dog owners caring for aged and ill animals (translated by the first author).

**Topic**<br>*Keywords and time (min)*	**Keywords**
Presentation (5)<br>*Of interviewer*<br>*Of the project*<br><br>*Consent to record*<br><br>*Outline of procedure*<br><br><br>*Opportunity to withdraw*<br><br>*Break*	Stine B Christiansen, PhD-student, University of Copenhagen, Bioethics.<br>Interview is part of PhD-project.<br>Concerns animal owner experiences when caring for aged and ill animals.<br>Happy to explain more after the interview.<br>Recording interview – will be anonymized.<br>Is it okay to record the interview?<br>I ask a number of questions and you answer what comes to your mind. No right or wrong answers.<br>May take notes – it is because there is something I would like to hear more about later in the interview.<br>If you think of something you find important, but I don't ask about it, you are very welcome to bring it up yourself.<br>If I ask about something you don't want to talk about, you can just say that you don't want to answer.<br>You can also stop the interview at any time.<br>If you want a break during the interview, just say so.

**Topics and keywords**	**Potential questions**
Changes in daily routines (20)<br>*Warm up*<br><br>*Routines regarding:*<br>*- care of the dog*<br>*- housekeeping and use of home*<br>*- work*<br>*- other commitments/interests*<br>*- social relations*<br>*Response from other people*	• Have you always had a dog? When did you get [the dog's name]?<br>• How did you find out that your dog was ill? Can you describe what happened from then till now/till you had to say good bye?<br>• What was a typical day like for you and your dog after it became ill? How was it different?<br>• Was there anything at home that you started to do differently or changed?<br>• Work/hobbies/friends?<br><br>• Do you remember any reactions from other people?
Experience of changes (20)<br>*Facing the diagnosis*<br>*Daily routines afterwards*<br>- *reaction to greater demand for care*<br>- *what was it like to have to….*<br>*Be accountable for choice of treatment*	• Can you tell me about the time when you were told that your dog had [the diagnosis]…..?<br>• Do you remember how you responded when you found out about the greater demands for care? What do you think about having to …? (give medicine, do extra cleaning, go to the vet (often)….)<br><br>• You mentioned some episodes, where people had said/asked about…Do you remember how you

- *reaction to people's comments*<br>*Veterinary visits*<br>- *a typical visit*<br>- *a special visit*<br>*The animal's and own welfare*<br>- *considerations about euthanasia*<br>- *kind of concerns*<br>- *reactions to concerns*<br>*The animal's death*<br>*Positive experiences*<br>- *have there been any*<br>- *something easier than expected*<br>- *any surprises*	responded, when people asked/commented…..?<br>• Can you describe a typical visit to the vet? Do you remember any visit in particular?<br>• Have you ever considered the possibility of euthanasia/Do you remember when you first considered the possibility of euthanasia?<br>• Did your thoughts revolve around anything in particular during this time? Did you worry about anything during this time?<br>• Can you tell me about the day that [the dog's name] died?<br>• Do you think there were any positive experiences in the process? Please explain….<br>• You mentioned earlier ……….., can you say some more about what happened then?<br>• Did anything turn out to be easier than you had expected?<br>• Did anything surprise you?
Importance of ressources (20)<br>*Economy*<br>- *considerations*<br>- *priorities*<br>*Knowledge*<br>- *about the medical condition*<br>- *experience giving medicine*<br>*Help on practical matters*<br>*Someone to talk to*<br>*Support from veterinary staff*	• Did you consider the financial aspects of the situation? How did you afford it?<br>• Did you know anything about [the medical condition] before?<br>• Did you have any experience caring for an ill/aged animal/give medicine?<br>• Was it difficult to….?/What do you think was the hardest part of ….? (an actual issue already mentioned/the whole process…) Was there anything that made/could have made ………easier?<br>• Did you have anyone to share the responsibility with?<br>• Who did you talk to about how it went?/Did you talk to the rest of the family/some friends/other dog owners/the vet about how it went?<br>• Do you think the vet/vets did/could have done something to help you through this?
Overall and good advice (10)<br>*Animal owners*<br>*Vets*	• What would you say to an animal owner facing the same situation?<br>• And to vets with clients facing the same situation?

Wrap up (5-10)<br>*Possible additions*<br>*Possible follow-up*<br>*Possible questions about the project*<br>*Thanks*	I don't have any more questions, is there anything you would like to add?<br>If you remember something later on that you think I should know, you can contact me and we may arrange a follow-up interview.<br>Do you have any questions about the PhD-project or the further processing of the information you have provided?<br>Thank you for your help. Handing over voucher.
